# Supplementary material for: Blood-based epigenome-wide analyses of chronic low-grade inflammation across diverse population cohorts
Source: Cell Genom. 2024 Apr 30;4(5):100544. doi: 10.1016/j.xgen.2024.100544 (PMC11099341; doi:10.1016/j.xgen.2024.100544)
Supplement: Document S1. Figures S1‒S10 [file mmc1.pdf]

Cell Genomics, Volume *4*

Supplemental information

Blood-based epigenome-wide analyses of chronic low-grade inflammation across diverse population cohorts

Robert F. Hillary, Hong Kiat Ng, Daniel L. McCartney, Hannah R. Elliott, Rosie M. Walker, Archie Campbell, Felicia Huang, Kenan Direk, Paul Welsh, Naveed Sattar, Janie Corley, Caroline Hayward, Andrew M. McIntosh, Cathie Sudlow, Kathryn L. Evans, Simon R. Cox, John C. Chambers, Marie Loh, Caroline L. Relton, Riccardo E. Marioni, Paul D. Yousefi, and Matthew Suderman

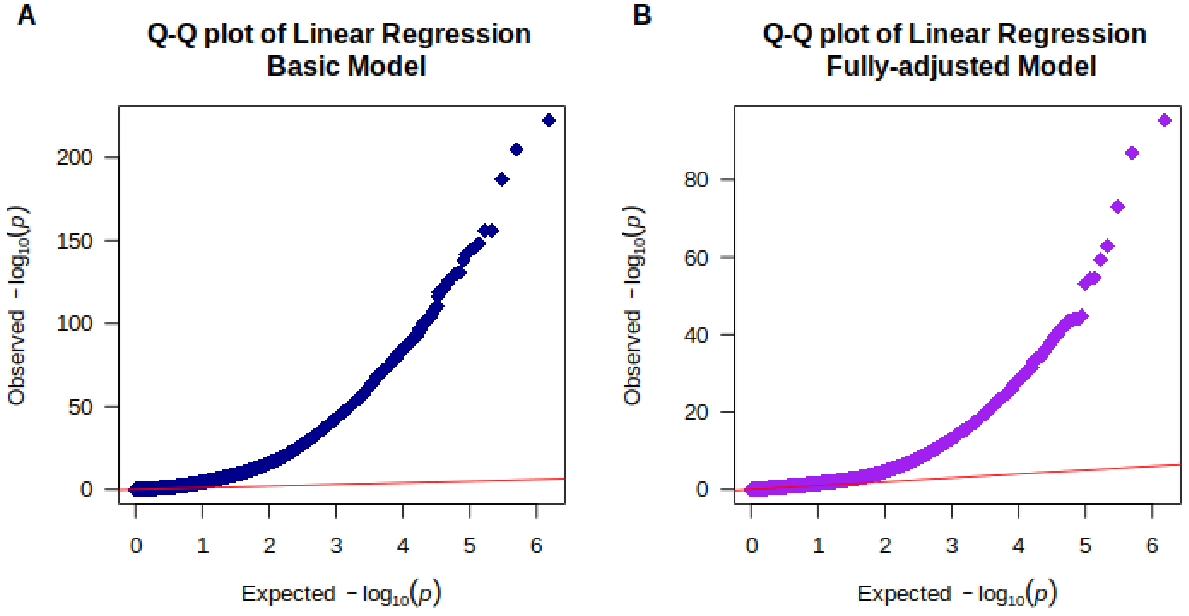

Figure S1. **Q-Q plots for the basic and fully-adjusted models in the linear regression EWAS, related to Figure 2.** EWAS, epigenome-wide association study.

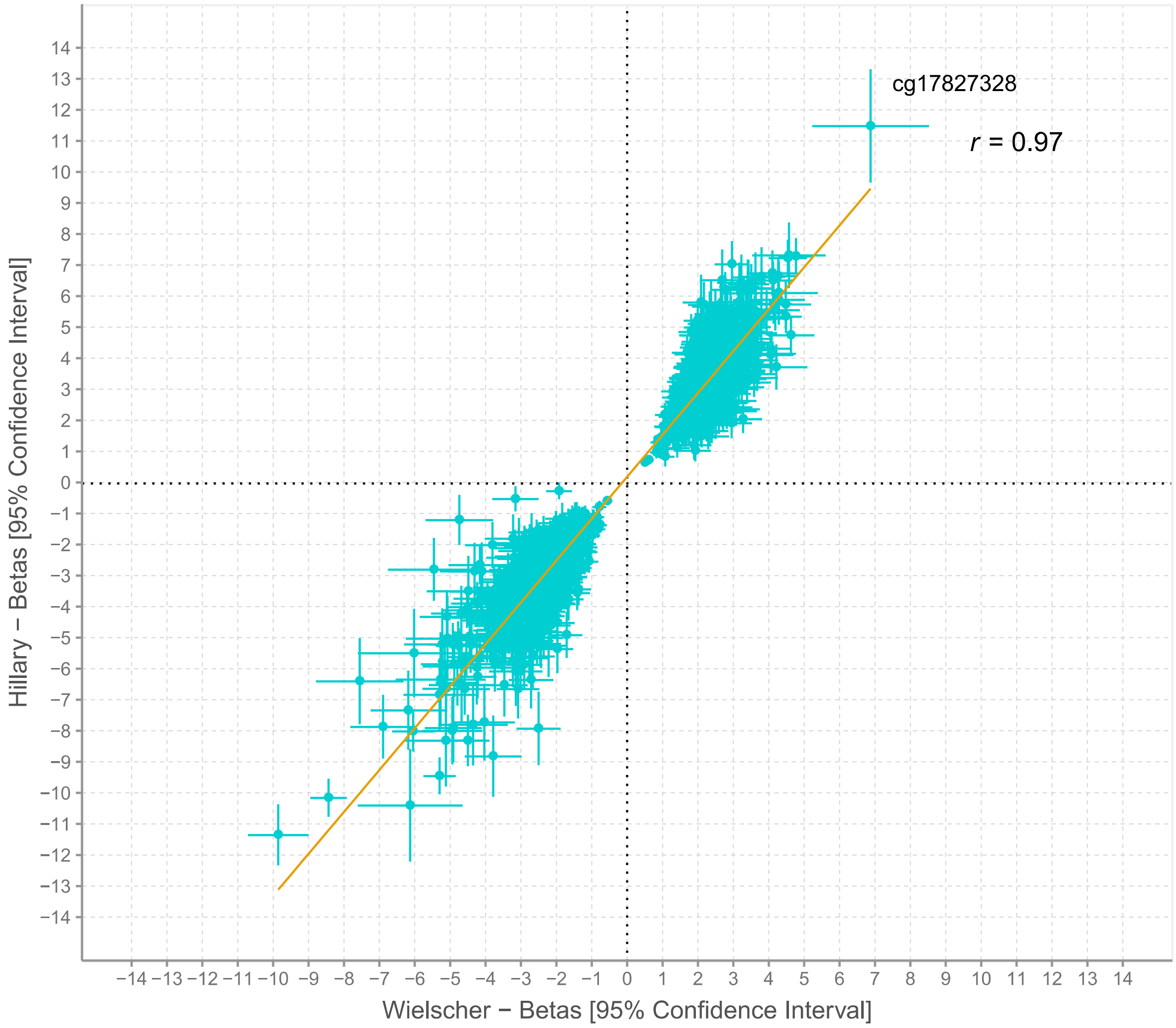

Figure S2. Correlation of effect sizes between epigenome-wide association studies by Wielscher *et al.* and the present study (Hillary *et al.*), related to Figure 2. Outputs from a basic model are shown, with matched analytical strategies between the two studies. Effect sizes for CpG sites that were significantly associated with blood CRP levels in the Wielscher *et al.* study (at $p < 3.6 \times 10^{-8}$) were compared against corresponding effects in the present study. There were 1,379 such CpG sites common to both studies that underpinned the correlation test. CpG, cytosine-phosphate-guanine dinucleotide; CRP, C-reactive protein.

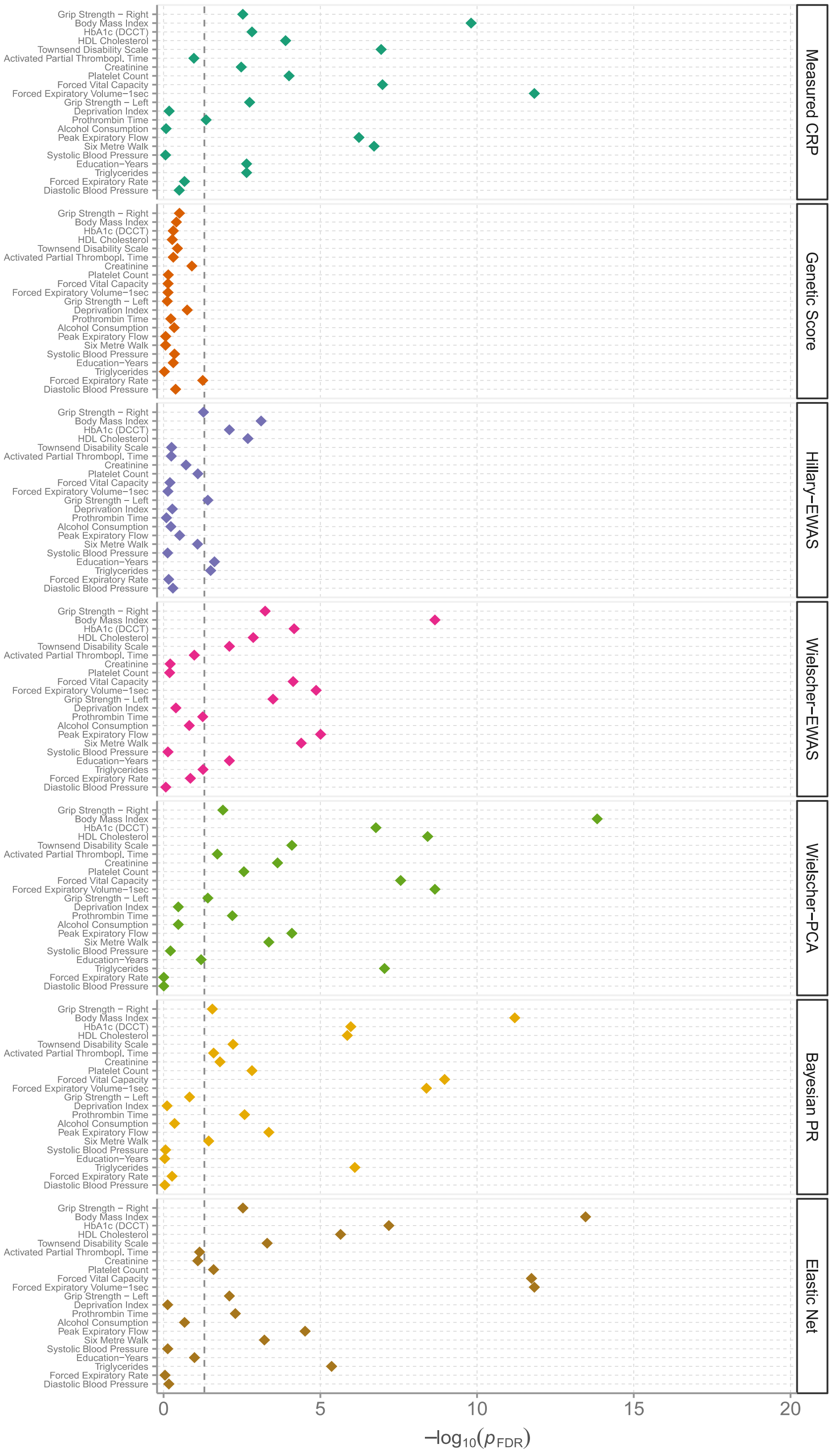

Figure S3. Association of 21 continuous cardiometabolic and lifestyle variables with measured CRP, genetic score for CRP and five DNAm predictors of CRP, related to Figure 4. CRP, C-reactive protein; DCCT; Diabetes Control and Complications Trial; DNAm, DNA methylation; EWAS, epigenome-wide association study; FDR, false discovery rate; HDL, high-density lipoprotein; PCA, principal component analysis; PR, penalised regression; Thrombopl., thromboplastin.

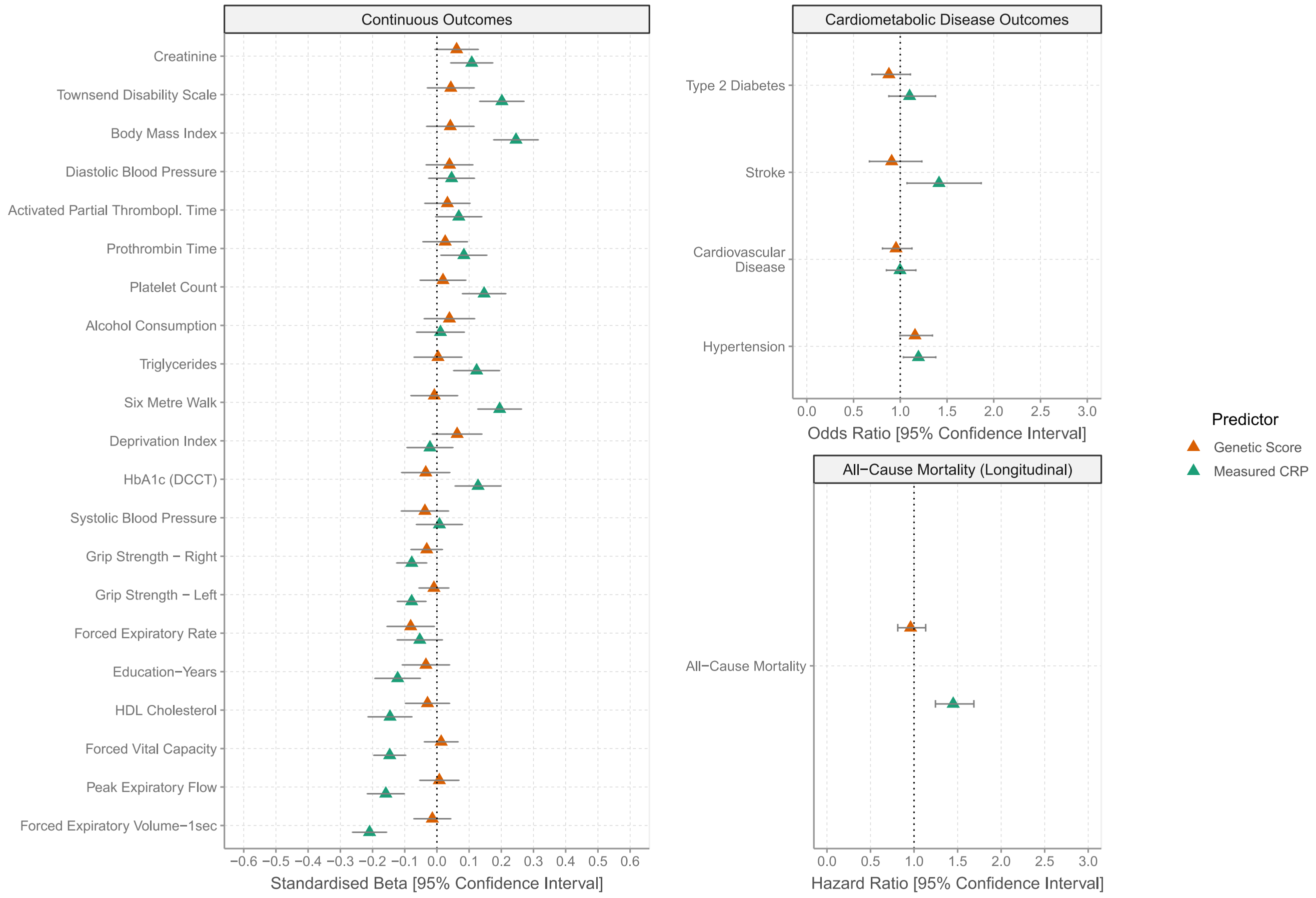

Figure S4. Associations of health outcomes with a genetic score for CRP or phenotypic CRP in the Lothian Birth Cohort 1936, related to Figure 4. CRP, C-reactive protein; DCCT; Diabetes Control and Complications Trial; DNAm, DNA methylation; HDL, high-density lipoprotein; Thrombopl., thromboplastin.

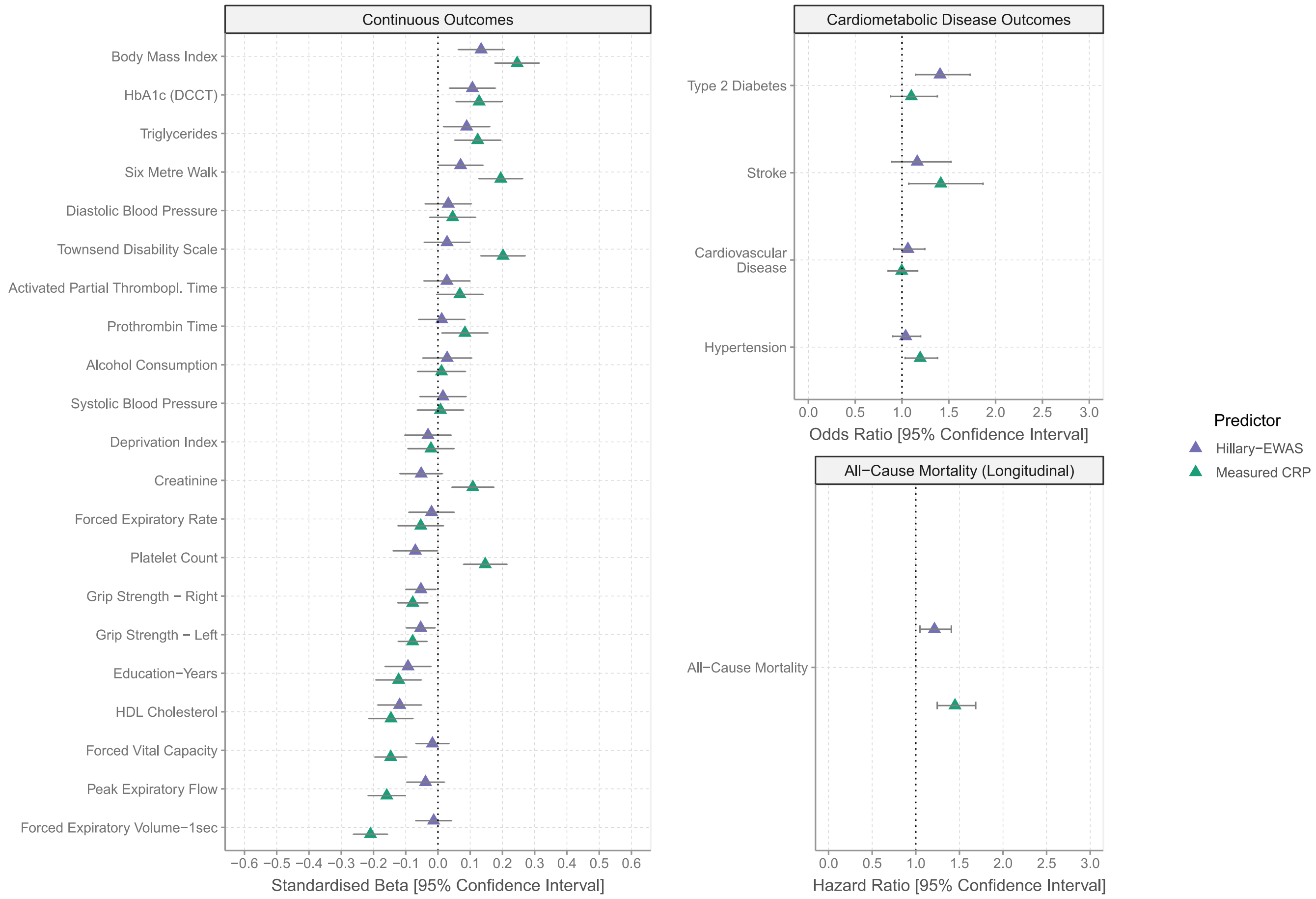

Figure S5. Associations of health outcomes with DNAm CRP (Hillary EWAS-based predictor) or phenotypic CRP in the Lothian Birth Cohort 1936, related to Figure 4. CRP, C-reactive protein; DCCT; Diabetes Control and Complications Trial; DNAm, DNA methylation; EWAS, epigenome-wide association study; HDL, high-density lipoprotein; Thrombopl., thromboplastin.

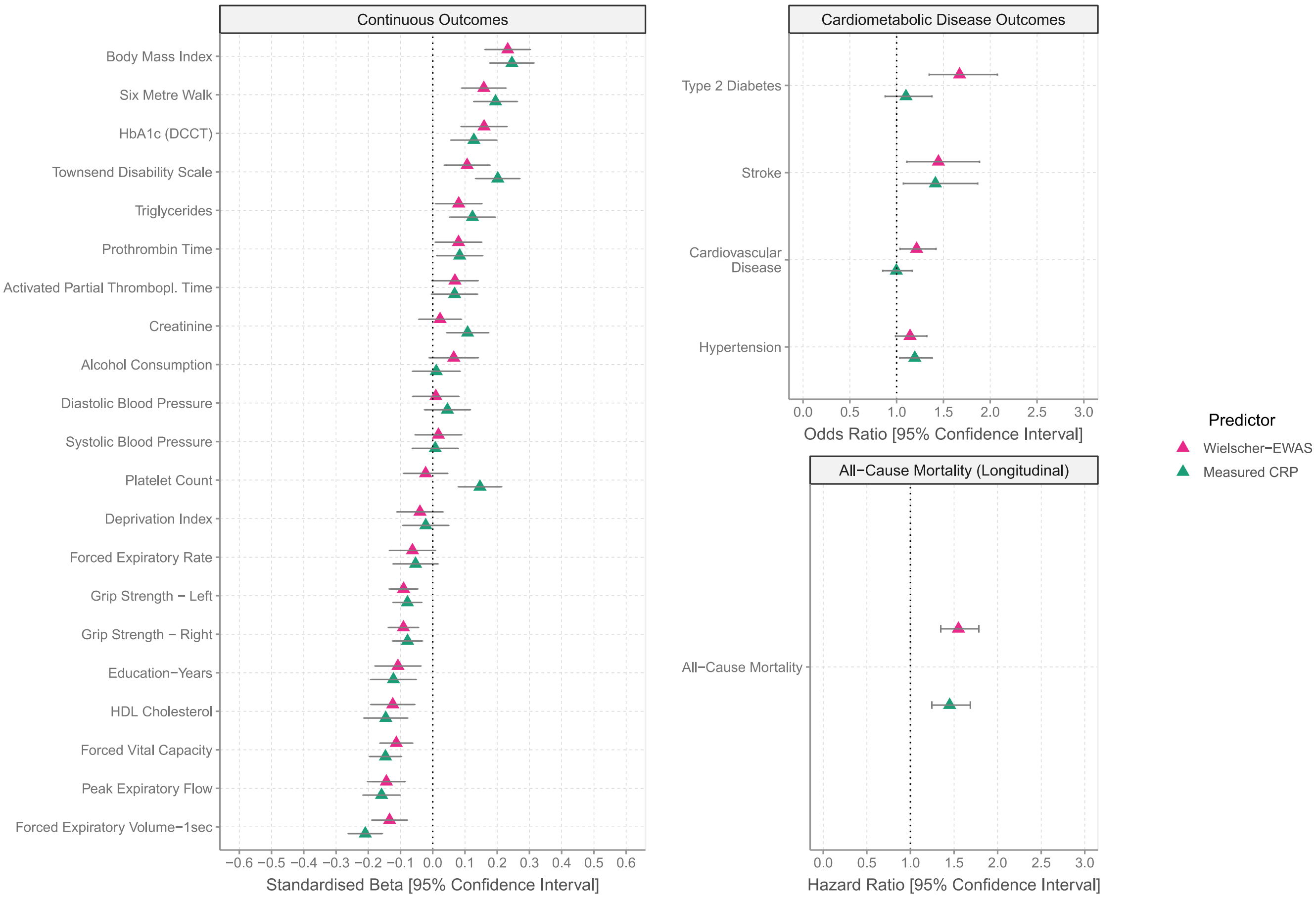

Figure S6. Associations of health outcomes with DNAm CRP (Wielscher EWAS-based predictor) or phenotypic CRP in the Lothian Birth Cohort 1936, related to Figure 4. CRP, C-reactive protein; DCCT; Diabetes Control and Complications Trial; DNAm, DNA methylation; EWAS, epigenome-wide association study; HDL, high-density lipoprotein; Thrombopl., thromboplastin.

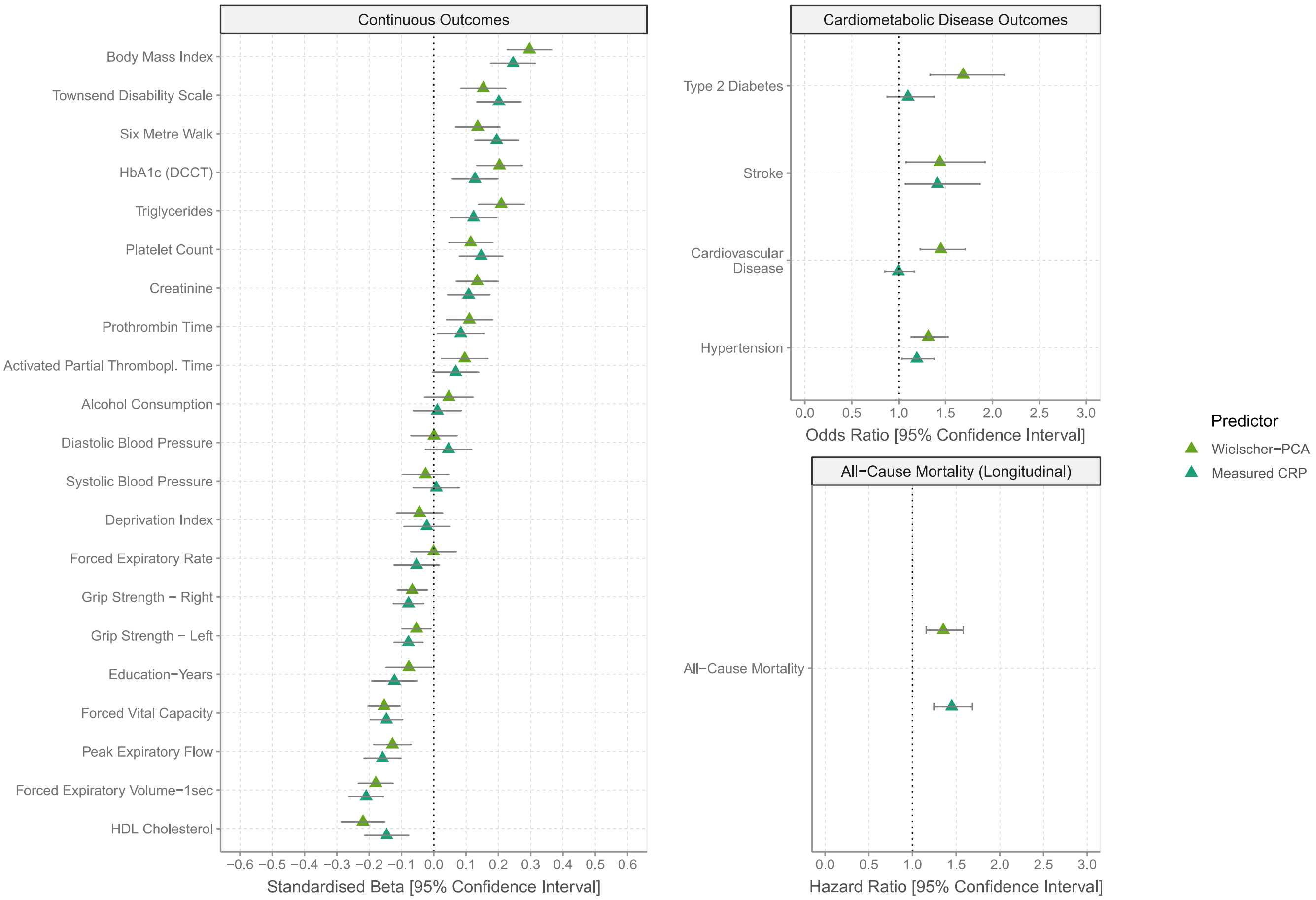

Figure S7. Associations of health outcomes with DNAm CRP (PCA-based predictor) or phenotypic CRP in the Lothian Birth Cohort 1936, related to Figure 4. CRP, C-reactive protein; DCCT; Diabetes Control and Complications Trial; DNAm, DNA methylation; HDL, high-density lipoprotein; PCA, principal component analysis; Thrombopl., thromboplastin.

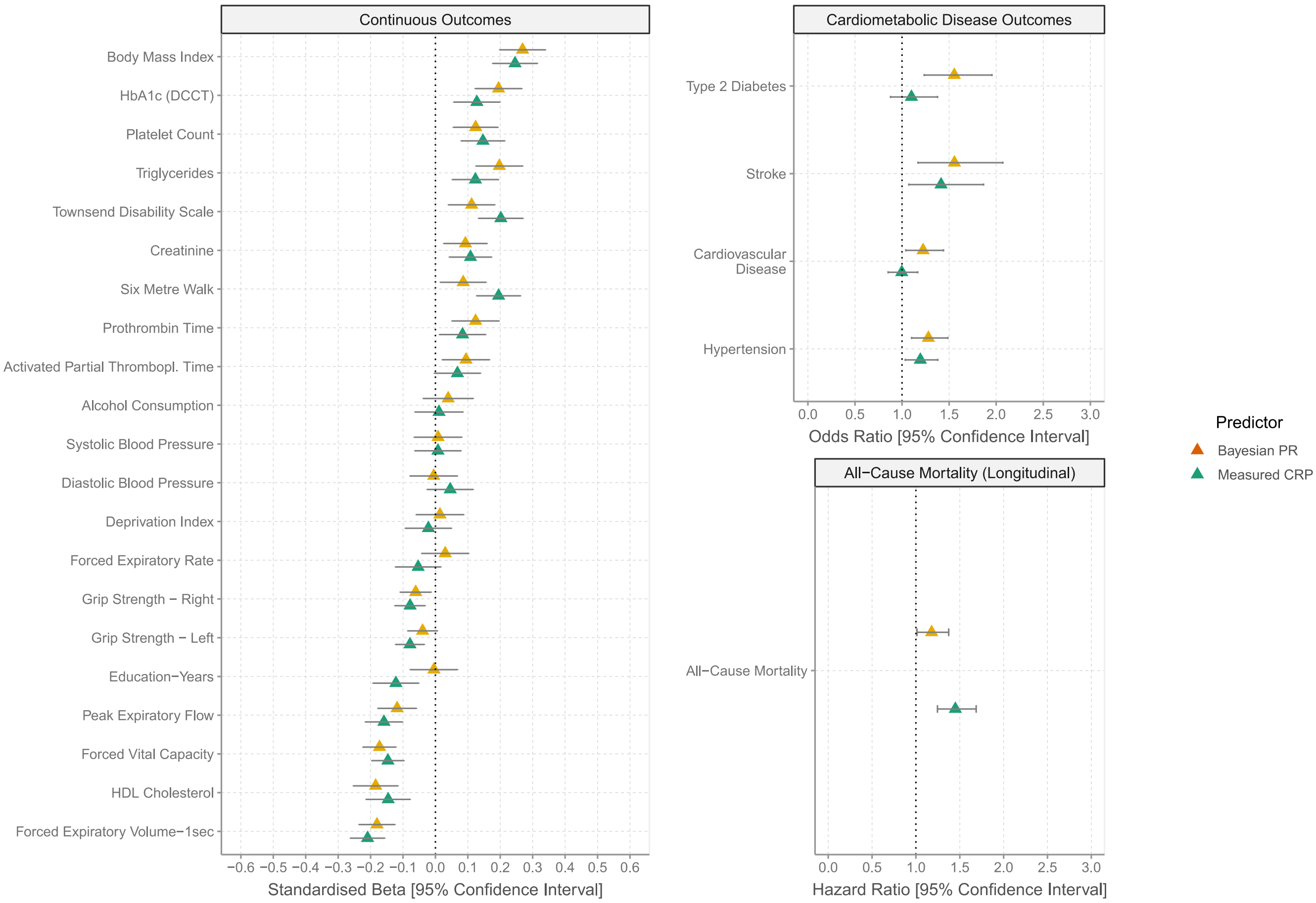

Figure S8. Associations of health outcomes with DNAm CRP (Bayesian PR-based predictor) or phenotypic CRP in the Lothian Birth Cohort 1936, related to Figure 4. CRP, C-reactive protein; DCCT; Diabetes Control and Complications Trial; DNAm, DNA methylation; HDL, high-density lipoprotein; PR, penalised regression; Thrombopl., thromboplastin.

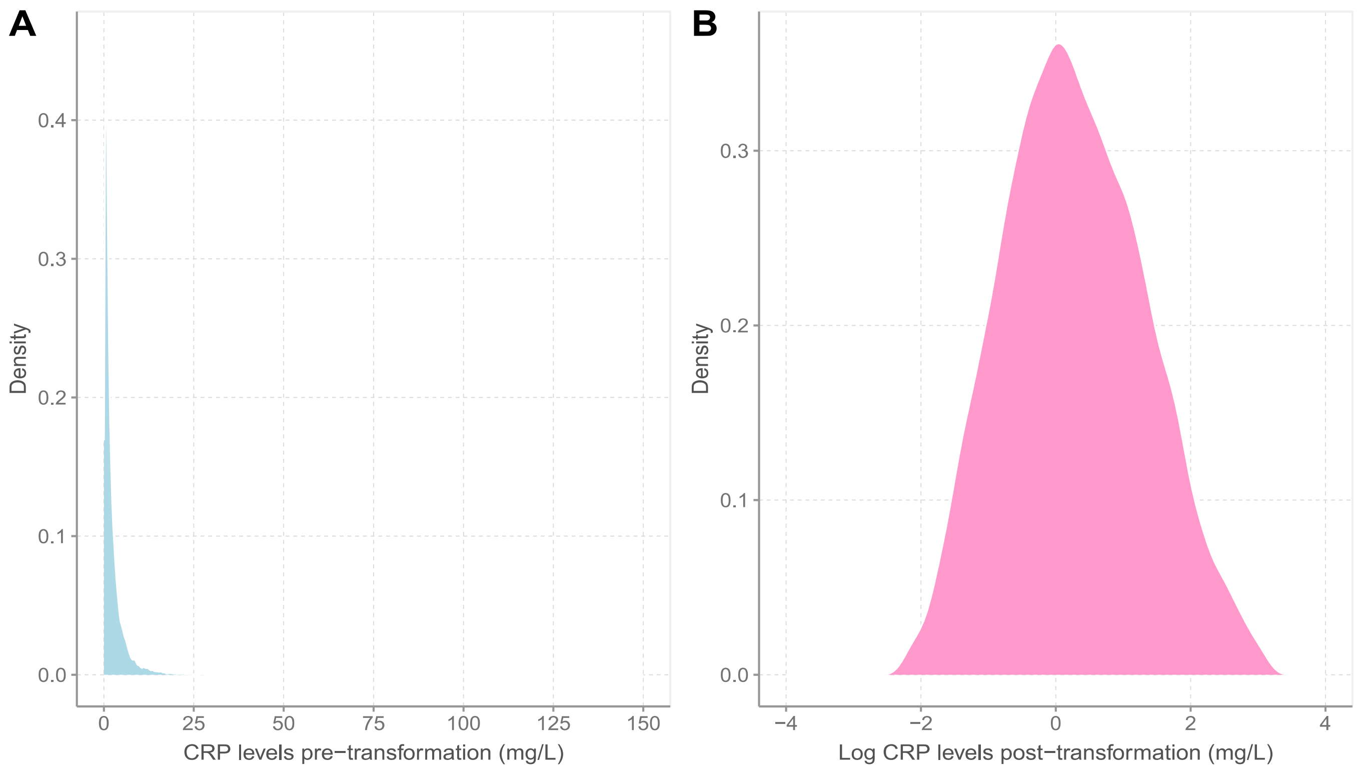

Figure S9. Distributions of CRP levels before and after statistical transformations in the training cohort, related to Figure 2. CRP levels were trimmed for outliers, which were defined as observations that were outside the median value ±4 times the standard deviation. CRP levels were also log-transformed to approximate a normal distribution and a constant of 0.01 was added to prevent undefined values. (A) shows values in Generation Scotland prior to these transformation steps. (B) shows values following statistical transformation. CRP, C-reactive protein.

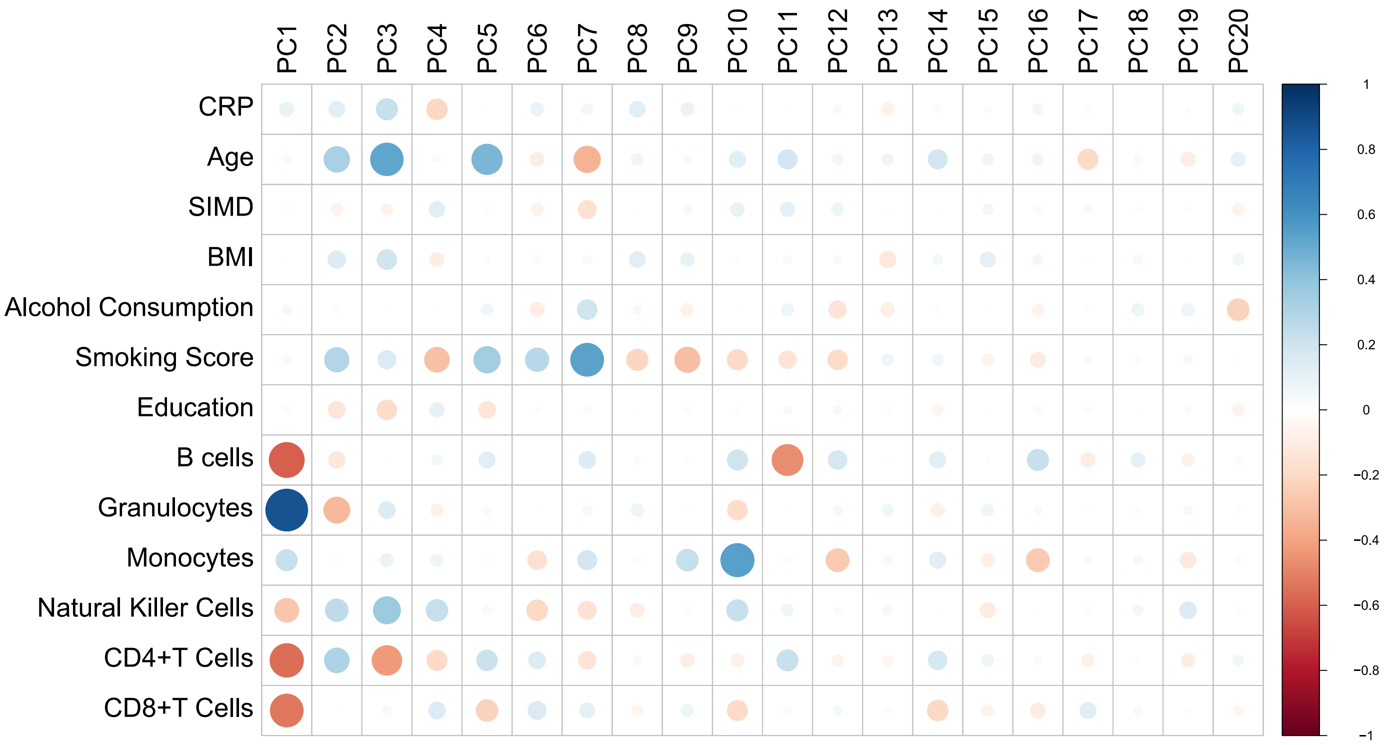

Figure S10. Correlation between the first 20 principal components on CRP-associated probes and continuous covariates in Generation Scotland, related to Figure 3. Principal component analysis was applied to a set of CRP-associated probes from Wielscher *et al.* in Generation Scotland (N=17,936). Only associations for the first twenty principal components are shown for clarity. BMI, body mass index; CRP, C-reactive protein; PC, principal component; SIMD, Scottish Index of Multiple Deprivation.
